# SPAI: an interactive platform for indel analysis

**DOI:** 10.1186/s12864-016-2824-x

**Published:** 2016-08-31

**Authors:** Mohammad Shabbir Hasan, Liqing Zhang

**Affiliations:** Department of Computer Science, Virginia Tech, Blacksburg, VA 24061 USA

## Abstract

**Background:**

Insertions and Deletions (Indels) are the most common form of structural variation in human genome. Indels not only contribute to genetic diversity but also cause diseases. Therefore assessing indels in human genome has become an interesting topic to the research community. This increasing interest on indel calling research has resulted into the development of a good number of indel calling tools. However, all of these tools are command line based and require expertise from Computer Science (CS) to execute them which makes it challenging for researchers from non-CS background.

**Methods:**

In this paper, we describe an interactive platform named SPAI which stands for Single Platform for Analyzing Indels.

**Results:**

Being a Graphical User Interface (GUI) tool, SPAI facilitates users to run several popular indel calling tools and perform several analyses on the indel calling results without knowing any command line programming.

**Conclusions:**

SPAI is written in Java and tested in Linux operating system.

## Background

Single Nucleotide Polymorphisms (SNPs) constitute the major portion of genetic variations that happen in human genome. However, recent studies show that insertion and deletions which are collectively known as indels also contribute to genetic diversity dramatically [1, 2]. Being a structural variant, indels can alter human phenotype and therefore can cause several kinds of diseases [3, 4]. For example, Cystic Fibrosis, one of the most common genetic diseases in humans, is caused due to the deletion of 3 base pairs (bps) which leads to the elimination of a single amino acid from the encoded protein [5]. Similarly insertion of base pairs in the DNA sequence results change in gene function that cause diseases like Fragile X Syndrome [6], Mendelian disorders [7], Haemophilia [8], Neurofibromatosis [9], Muscular Dystrophy [10], and Cancer [11, 12]. In addition to causing diseases, indels within the promoter region influence gene expression and can be used to explain the difference in gene expression observed in different human [13] which apparently brings the use of indels as genetic markers in natural population [14].

With the introduction of Next Generation Sequencing (NGS) technology, now it is possible to sequence human genome at an unprecedented rate [1] and whole genome sequencing (WGS) is now possible at an individual level [15–19]. Whole genome sequencing has revealed numerous genetic variations that were not previously reported [20] and these variation profiles can also be used to predict ancestor’s traits such as height, weight, appearance, and intelligence [1]. Therefore the idea of predicting the future health of individuals to design personalized medicine is rapidly approaching. However, accurate detection of genetic variation at an individual level is a key challenge in evolutionary genomic research. To accept this challenge, fortunately, a good number of indel calling tools have been developed so far [21, 22].

In recent time, many indel calling tools have been developed which are publicly available as well as popular among the researchers. Some of these tools include Genome Analysis Tool Kit (GATK) [23, 24], VarScan [25, 26], Pindel [27], SAMtools [28], Dindel [29], Platypus [30], P-Dindel [21], SV-M [31], Stampy [32], PEMer [33], Hydra [34], BreakDancer [35], FreeBayes [36], and indelMINER [37]. A close look at these tools reveals that all of them are command line based [38]. Command line tools are very much useful for batch processing and they require a certain level of expertise from Computer Science (CS). Therefore, researchers from non-CS background such as Biology, Chemistry, and Microbiology, doing research on evolutionary genomics, often find it difficult to execute and explore different features of the tools by changing different parameters through command line.

Research by several usability labs reveal that the usability of a product can be significantly improved through the use of Graphical User Interface (GUI) [39] and providing the user with GUI for command line based bioinformatics tools became a success previously [40].

To come up with a solution to the usability problem of the indel calling tools, here we describe SPAI (Single Platform for Analyzing Indels). SPAI provides the user with a complete platform for indel research. In addition to running popular indel calling tools, through SPAI, user can also download alignment files (BAM files) form the 1000 Genome Project [41] to be used as input to these tools. Moreover, user can get coverage information of these alignment files and see the alignment files in a tabular format. In SPAI, user can also see the indel calling results in a tabular format to get a better insight of the called indels. For downstream analysis, SPAI lets the users to compare the results from various tools and visualize those comparisons using graphs and charts. Being an interactive tool, therefore, SPAI lets the user to perform necessary works of indel research without having any prior knowledge of command line programming.

## Methods

SPAI which is written in Java comes with several features that are briefly described below.

### Running different indel calling tools from GUI

**Fig. 1:**
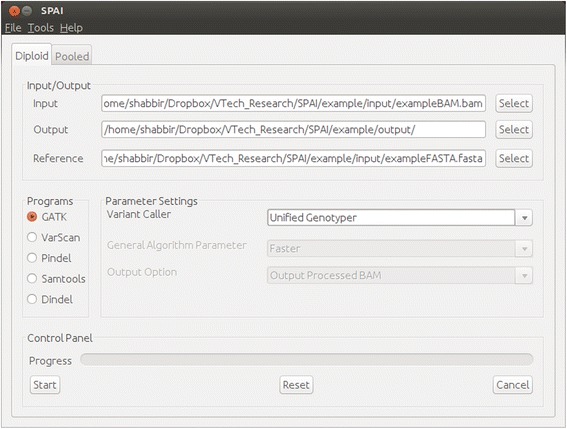
Main window of SPAI

Existing indel calling tools can be divided into four major categories: alignment based methods, split read methods, paired end read mapping methods, and haplotype based methods [22]. In the current version of SPAI, we include tools from two categories: alignment based methods and split read methods. From the alignment based methods category, SPAI includes GATK Unified Genotyper, VarScan, Dindel, and SAMtools. It also includes Pindel which belongs to the split read method category. When the user installs SPAI, these tools are also installed automatically. In the next release of SPAI, the following tools will be added: GATK Haplotype Caller, Platypus, FreeBayes, and indelMINER. Other tools from different categories will be added as the development of SPAI proceeds. Figure 1 shows the main GUI of SPAI. As we can see from this Figure, user just needs to specify the inputs (alignment file and reference sequence file), output file location and which tool to run. Usually SPAI runs the selected indel calling tool using its default settings. However, SPAI allows advanced user to change different parameter of each of the tools and run those tools based on that settings.

Some of the programs require huge processing power to generate results in a reasonable time. Although the current version of SPAI is desktop based, we are now working on moving the processing step in a cloud based service so that enough processing power can be provided and in that case the computation time will no longer depend on the configuration of user’s computer.

### Download alignment files from the 1000 Genomes project

**Fig. 2:**
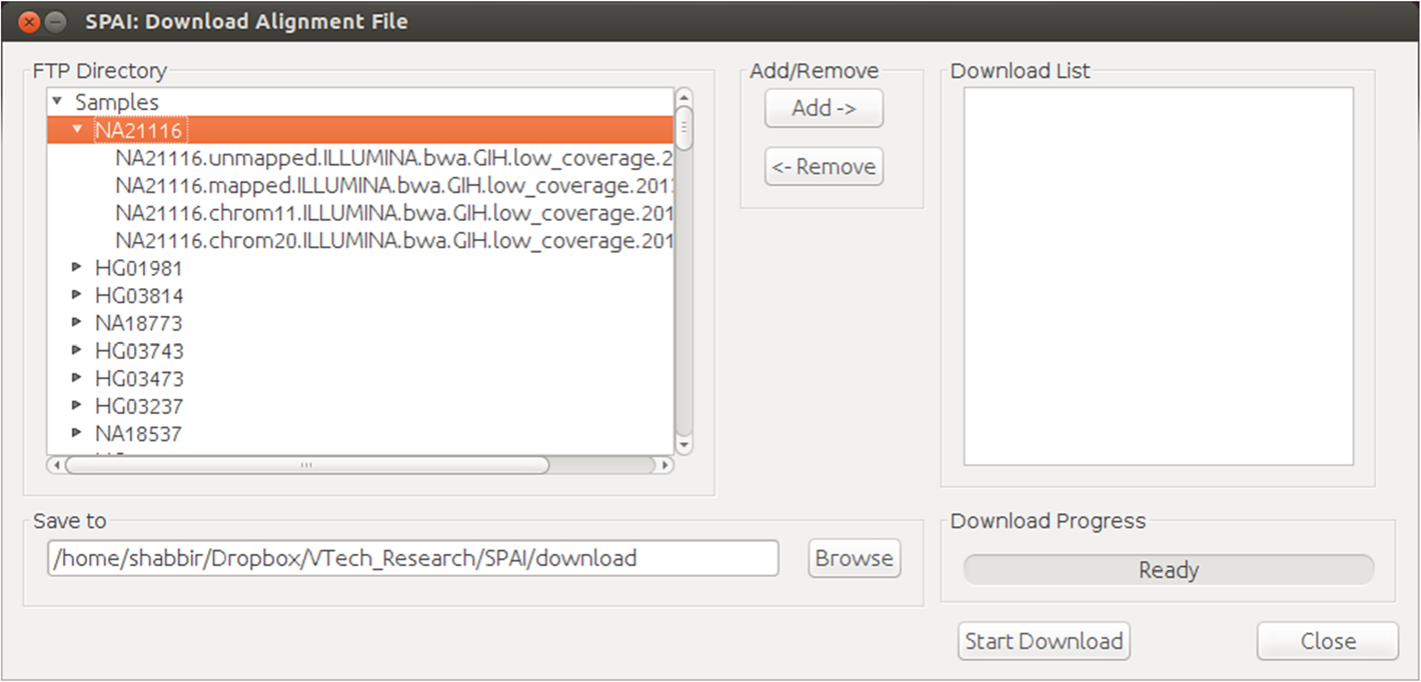
Downloader window of SPAI

Input to the existing indel calling tools is the sequence alignment file which is usually available in BAM format. In most of the cases, the size of the BAM files is huge which can’t be downloaded using the conventional downloader. To assist user in this case, an efficient downloader is integrated in SPAI which allows user to download single as well as multiple BAM files from the FTP server of the 1000 Genomes project. As shown in Fig. 2, the left panel of the downloader window shows the list of all human samples currently available in the 1000 Genomes project. From this list the user needs to select for which human and for which chromosome the alignment file is needed. After the selection is done, the file is added to the “Download List” as shown in the right panel of the downloader window. User can add multiple files to the downloader list or remove file from the list. After the selection is done, when the user hits the “Start Download” button, SPAI starts downloading the BAM file(s) and store it in the location specified by the user.

Sometimes it is inconvenient to store large BAM files in the local directory of the user’s computer. Therefore, in the future release, SPAI will store the URLs of the alignment files in a text file in user’s computer and will save the alignment file in a cloud based storage. Therefore while specifying the inputs, SPAI will allow user to put the URL of an alignment file instead of the physical location of that alignment file. It will also allow user to put external link to alignment files stored in different location other than the FTP server of the 1000 Genomes Project and in that case SPAI will fetch the alignment file and save it temporarily to the cloud based storage to be used during the execution time.

### Comparing the results of different indel calling tools

**Fig. 3:**
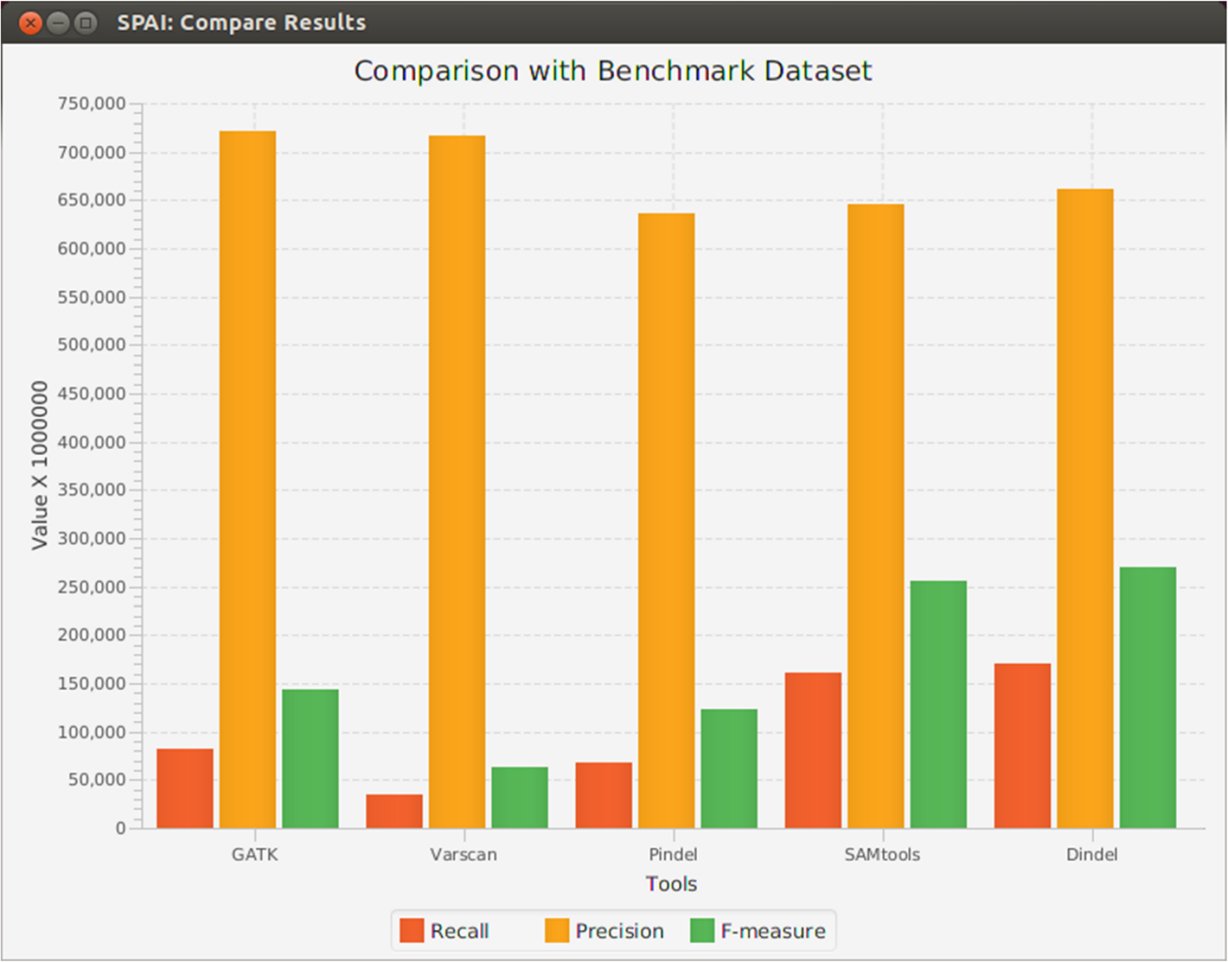
Comparing the results of different tools with benchmark dataset

User can compare the indel calling results produced by different tools which is a really useful feature for downstream analysis. In SPAI we provided a benchmark dataset [42] which contains 2 million small and large (length varies from 1 bp to 10,000 bps) indels found in the 24 chromosomes of 79 diverse humans. This dataset is considered to be the most reliable for indels in human genome and has been used as “gold standard” in other studies [22, 43, 44]. From the VCF (Variant Call Format) files that are generated by the tools as output, SPAI calculates the recall, precision, and F-measure of each tool after comparing their results with the benchmark dataset. User can see the comparisons as Graphs (shown in Fig. 3) which is really helpful to get an insight about the performance of the tools. Moreover, for the comparison purpose, SPAI allows user to supply results (in VCF format) from other tools that are not included in SPAI. This is really useful if the user want to assess the performance of a newly developed tool by comparing its result with existing tools as well as with “gold standard” indels.

### Displaying the alignment files and indel calling results in tabular format

**Fig. 4:**
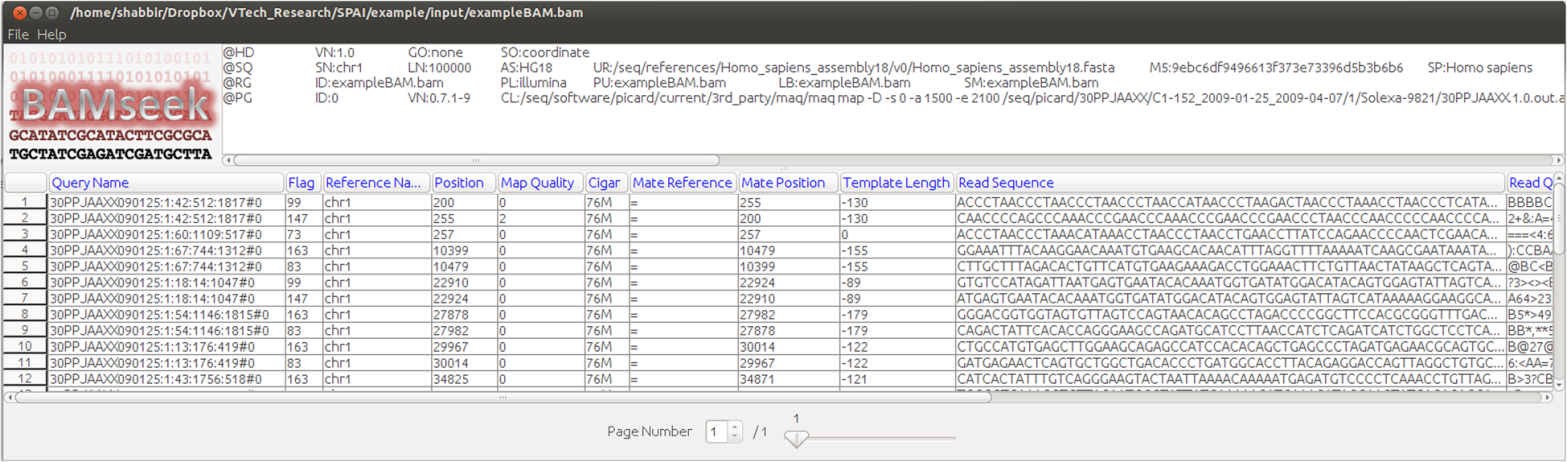
Tabular display of a BAM file

Sometimes alignment files contain useful information about the reads (such as mapping quality, CIGAR string, type of the read etc.). However, the standard format of the alignment files is BAM which is a binary format and therefore, can’t be opened using a text editor. Although SAMtools can convert BAM file to text format (SAM format), it is not convenient to open large SAM files using a text editor. To solve this problem, in SPAI, we include a third party tool called BAMSeek [45]. It can show large BAM files in a tabular format and user can get useful information about the alignment by hovering mouse to the corresponding column of the table. Similarly BAMSeek can also display large VCF files (output of the indel calling tools) in a tabular format from where user can get insight about the called indels. Figure 4 shows the tabular display of a BAM file.

### Determining the depth of coverage

Depth of coverage is the average number of reads that represent a given nucleotide in the sequence. In most cases, high depth of coverage is desired for calling indels confidently [22]. SPAI allows users to determine whether an alignment file should be used as input for indel calling by calculating the coverage of that alignment file.

### Future work

SPAI is an on-going project and under active development. In future release, we plan to move the indel calling steps of the tools to a cloud based service which will completely reduce the computational burden of user’s computer. Moreover, the future release will not require the user to download large alignment files. Instead, user will supply URL to the alignment file not only from the FTP server of the 1000 Genomes project but also from other sources and SPAI will fetch the file and produce result in the cloud based service. We will also let the user to open account in the cloud based service and the user’s previously obtained results will be stored in the account so that these results can be used later instead of running the tools again. We plan to keep including newly developed indel calling tools and the next release will include some highly used tools such as GATK Haplotype Caller, Platypus, FreeBayes, and indelMINER. Moreover, we will also add utility tools such as UPS-indel - a tool to find ambiguous indels [46]. In addition to that we also plan to include tools that display the effect of a list of indels in coding and non-coding regions. Batch processing feature will also be added which will allow the user to perform multiple tasks simultaneously.

## Results and Discussion

In this paper we addressed two research questions that are given below:

## What is the necessity of creating a GUI for existing indel calling tools?

Researchers heavily depend on existing command line programs for calling indels from their dataset as well as for downstream analysis to the problems of their research domain. Since most of these tools are very popular and widely used by the research community, the question which may arise is why don’t we just keep these tools as they are right now? From our experience of working on indel projects, we realized that to explore different features of these existing tools by changing parameters and/or by changing inputs, users need to write the command every time. This needs some expertise of command line programming. Moreover exploring tools by writing command every time causes lack of usability of these tools. To solve this usability problem and to overcome the requirement of command line programming expertise, we designed SPAI, a Graphical User Interface (GUI) based tool. Being a GUI based tool, SPAI allows users to explore the features of existing indel calling tools just by selecting input through a regular file browser and setting parameters by writing it in a text box. This not only saves time required for writing commands, but also gives users a better user experience. Moreover, since SPAI includes multiple indel calling tools in the same platform, user can run multiple tools at the same time for same inputs without writing a single line of command.

## How SPAI can help in downstream analysis of indels?

After calling indels, the next step is the downstream analysis of the called result. The research question that we addressed is how SPAI can help in this context? SPAI comes with a list of known indels [42] for human genome which has been used as benchmark dataset by many researchers. After importing indel calling results from different tools, SPAI compares those indels with the above mentioned benchmark dataset. SPAI produces the comparison results in graphical format and also provides statistics such as recall, precision, and F-measure. This feature of SPAI allows users to assess the performance of the indel calling tools based on these matrices. Moreover, user can also supply their own benchmark dataset and list of indels produced by their own tool. Since SPAI produces graphs and charts with performance comparison matrices, user can easily assess the performance of their tools without doing these comparisons by themselves. This also saves time and ensures better user experience.

## Conclusions

Indels constitute the most common form of structural variation in human genome and have been found to be responsible in causing diseases by abolishing gene functions. In addition to that, indels can influence human traits and gene expression and therefore can be used as genetic marker. All these statements lead to the necessity of variant profiling which should be achievable as whole genome sequencing at individual level is now possible because of Next Generation Sequencing (NGS) technology. A good number of indel calling tools have been developed that can be used for variant profiling purpose. However, all of these are command line based which require certain expertise from Computer Science (CS). As evolutionary genomics is a multi-disciplinary research area, people from non-CS background are also involved in indel calling research and should be able to use these tools without prior knowledge of command line programming. Here we introduce SPAI (Single Platform for Analyzing Indels) which provides user with an interactive platform to use popular indel calling tools using a user friendly GUI and perform different analyses without knowing any command line programming. We believe that people especially from non-CS background will find SPAI really useful while performing their indel calling research.
